# Architecting Braided Porous Carbon Fibers Based on High‐Density Catalytic Crystal Planes to Achieve Highly Reversible Sodium‐Ion Storage

**DOI:** 10.1002/advs.202104780

**Published:** 2022-04-26

**Authors:** Chuanqi Li, Zhijia Zhang, Yuefang Chen, Xiaoguang Xu, Mengmeng Zhang, Jianli Kang, Rui Liang, Guoxin Chen, Huanming Lu, Zhenyang Yu, Wei‐Jie Li, Nan Wang, Qin Huang, Delin Zhang, Shu‐Lei Chou, Yong Jiang

**Affiliations:** ^1^ State Key Laboratory of Separation Membrane and Membrane Processes Tianjin Municipal Key Laboratory of Advanced Fiber and Energy Storage School of Materials Science and Engineering School of Electronic and Information Engineering School of Mechanical Engineering Tiangong University Tianjin 300387 China; ^2^ Institute for Carbon Neutralization College of Chemistry and Materials Engineering Wenzhou University Wenzhou Zhejiang 325035 China; ^3^ School of Materials Science and Engineering University of Science and Technology Beijing Beijing 100083 China; ^4^ School of Materials Science and Engineering Tianjin University Tianjin 300072 China; ^5^ Ningbo Institute of Materials Technology and Engineering Chinese Academy of Sciences Ningbo 315201 China; ^6^ Institute for Superconducting and Electronic Materials University of Wollongong Wollongong NSW 2522 Australia; ^7^ China Electronics Technology Group Corporation No. 46 Institute CETC JH(Tianjin) Semiconductor Material Co. Ltd. Tianjin 300220 China; ^8^ Guangdong Institute of Semiconductor Industrial Technology Guangdong Academy of Sciences Guangzhou 510651 China

**Keywords:** anode materials, catalytic active (111) planes, chemical vapor deposition, porous carbon fibers, sodium‐ion batteries

## Abstract

Carbonaceous materials are considered strong candidates as anode materials for sodium‐ion batteries (SIBs), which are expected to play an indispensable role in the carbon‐neutral era. Herein, novel braided porous carbon fibres (BPCFs) are prepared using the chemical vapour deposition (CVD) method. The BPCFs possess interwoven porous structures and abundant vacancies. The growth mechanism of the BPCFs can be attributed to the polycrystalline transformation of the nanoporous copper catalyst in the early stage of CVD process. Density functional theory calculations suggest that the Na^+^ adsorption energies of the mono‐vacancy edges of the BPCFs (−1.22 and −1.09 eV) are lower than that of an ideal graphene layer (−0.68 eV), clarifying in detail the adsorption‐dominated sodium storage mechanism. Hence, the BPCFs as an anode material present an outstanding discharge capacity of 401 mAh g^−1^ at 0.1 A g−1 after 500 cycles. Remarkably, this BPCFs anode, under high‐mass‐loading of 5 mg cm−2, shows excellent long‐term cycling ability with a reversible capacity of 201 mAh g^−1^ at 10 A g^−1^ over 1000 cycles. This study provided a novel strategy for the development of high‐performance carbonaceous materials for SIBs.

## Graphical abstract

Braided porous carbon fibres (BPCFs) present interwoven structures and abundant vacancies, originating from high‐density carbon “fiber seeds” induced by high‐density catalytic activity (111) planes. The BPCFs anode shows excellent cycling ability with a reversible capacity of 201 mAh g^−1^ at 10 A g^−1^ over 1000 cycles.

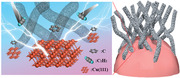

## 1 Introduction

Sodium‐ion batteries (SIBs), one of the alternatives to lithium‐ion batteries (LIBs), have promising commercial application prospects owing to abundant sodium resources and the similarity of their working mechanism to that of LIBs. However, the large sodium ion radius limits further improvement in the barrier performance, posing a challenge in designing novel electrode materials.^[^ ^1^ ^]^ Currently, carbonaceous materials^[^ ^2^ ^]^ such as hard carbon,^[^ ^3^ ^]^ soft carbon, carbon nanofibers (CNFs),^[^ ^4^ ^]^ and graphite^[^ ^5^ ^]^ are promising anode materials for SIBs because of their excellent electrical conductivity, low cost, and environmental friendliness.^[^ ^6^ ^]^ Although these carbonaceous anode materials have achieved remarkable results in recent years,^[^ ^7^ ^]^ there still exist difficulties in their practical applications, such as layer spacing modulation,^[^ ^8^ ^]^ micro‐nanostructure design,^[^ ^9^ ^]^ and a high commercialization cost. The sodium storage mechanism of carbonaceous materials has been generally recognized to involve the following: 1) a high‐potential sloping region capacity corresponding to Na^+^ adsorption and intercalation^[^ ^10^ ^]^ and 2) a low‐voltage plateau region related to pore filling.^[^ ^11^ ^]^ Thus, construction of appropriate defects and interlayer distances can remarkably improve the sodium storage capacity of carbonaceous materials.

Among carbonaceous materials, one dimensional CNFs (1D CNFs) with a large specific surface area and porosity present excellent sodium storage performance.^[^ ^12^ ^]^ Chemical vapor deposition (CVD) is a simple, low‐cost, controllable, and adjustable method for fabricating 1D carbon nanomaterials.^[^ ^13^ ^]^ The catalytic growth mechanism of CNFs mainly includes a “tip‐based model” and a “base growth model.”^[^ ^14^ ^]^ Particularly, the types and shapes of catalyst particles significantly affect the morphologies and structures of CNFs.^[^ ^15^ ^]^ Yang et al. reported that the preparation of CNFs can be controlled by modulating the morphology of copper nanoparticles as well as the adsorption of C_2_H_2_ on Cu (111).^[^ ^16^ ^]^ Additionally, there are many studies on methods to improve the sodium storage capacity of CNFs. Wen et al. reported an excellent high capacity of 148 mAh g^−1^ at a high current density of 10 A g^−1^ for nitrogen‐doped CNFs with interwoven nanochannels.^[^ ^9a^ ^]^ The Pint group reported that defect‐containing helical CNFs on 3D foams can present a sodium storage capacity exceeding 280 mAh g^−1^ at a moderate rate of 100 mA g^−1^ with a stable cycling performance over 200 cycles.^[^ ^17^ ^]^ However, the mechanism of high‐efficiency sodium storage based on the catalytic growth model of CNFs is still inconclusive.

In this study, braided porous carbon fibers (BPCFs) with dense vacancies and interwoven structures were synthesized by a one‐step in situ catalytic CVD method. The high‐density catalytically active (111) planes on the surface of a nanoporous multicrystal copper were found to preferentially adsorb carbon (C) atoms, forming “fiber seeds.” As the fibers grow, adjacent “fiber seeds” intertwine and form the framework for BPCFs. Concurrently, high‐density vacancies are formed during the process of carbon “fiber seeds” winding. The deposited carbon yield was evaluated as 140% after growth for 60 min. Consequently, the BPCFs anode presents a high specific capacity of 401 mAh g^−1^ at a current density of 0.1 A g^−1^ over 500 cycles. Meanwhile, the BPCFs anode shows remarkable cycling stability and the capacity remained 201 mAh g^−1^ after 1000 cycles with a high‐mass‐loading of 5 mg cm^−2^ at a high current density of 10 A g^−1^ used in SIBs. Based on the developed understanding of the growth mechanism of the CNFs, this novel strategy for regulating sodium storage offers a route for designing high‐performance carbonaceous sodium storage materials.

## 2 Results and Discussion

**Figure 1:**
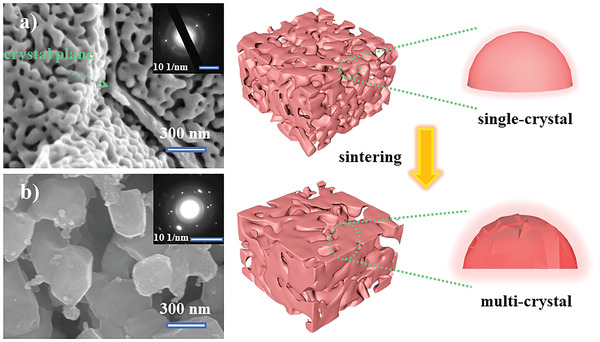
SEM images and schematic of a) 3D NPC and b) nanoporous multicrystal copper after sintering.

**Figure** **1** shows a schematic of the fabrication process of the nanoporous multicrystalline copper catalyst used in this study. A 3D nanoporous copper (3D NPC) with a 40 nm uniform ligament was obtained by chemical corrosion of Cu_30_Mn_70_ (at%) alloy in HCl solution (0.025 mol L^−1^). This process has many advantages such as easy preparation, low cost, and good feasibility (see Figures S1–S3, Supporting Information). The composition of the NPC was measured to be ≈92.41 at% Cu, ≈5.24 at% O, and ≈2.35 at% Mn, by energy dispersive X‐ray spectroscopy (Figures S1 and S2, and Table S1, Supporting Information). The presence of a small amount of Mn suggests that the nanoporous structure is mainly formed by the selective leaching of Mn from the Cu_30_Mn_70_ alloy, and the appearance of O reflects a slight oxidation of the 3D NPC. Figure 1a shows the presence of nanocopper ligaments in single‐crystal grains. The corresponding selected area electron diffraction (SAED) pattern further shown in the upper right inset in Figure 1a verifies this characteristic. However, the high growth temperature (600 ℃) causes coarsening of the copper ligaments. The coarsened copper ligaments exhibit a multicrystal structure, which is confirmed by scanning electron microscopy (SEM) and SAED (Figure 1b). This structure is expected to expose additional high‐catalytic activity (111) planes on each ligament during the growth process of the CNFs.^[^ ^18^ ^]^

**Figure 2:**
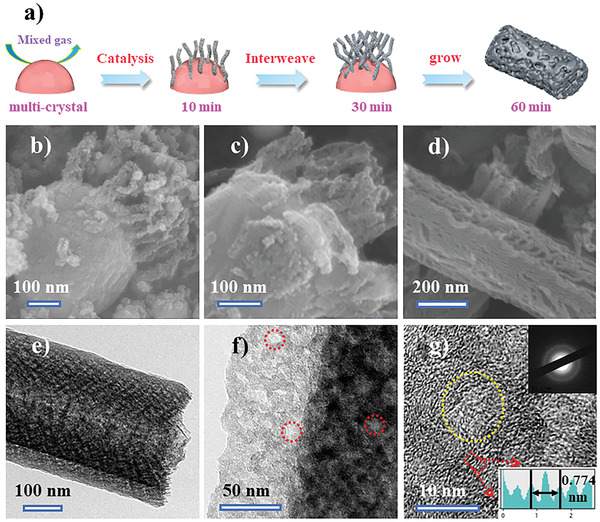
a) Schematic of growth process of BPCFs. SEM image of carbon fibres after b) 10 min, c) 30 min, and d) 60 min. e,f) TEM images of BPCFs at different magnifications, g) HRTEM image of BPCFs (insets: SAED pattern).

**Figure** **2**a illustrates the growth process of the BPCFs. As a catalyst, the synthesized nanoporous multicrystal copper with ligament sizes of 250–300 nm exposes high‐density catalytically active crystal planes. As shown in Figure 2b, after 10 min of growth, carbon “fiber seeds” are uniformly dispersed on the surfaces of the catalyst particles, indicating that the “fiber seeds” prefer to grow on the high‐catalytic activity (111) planes. Concurrently, Figure S4a (Supporting Information) shows the crystalline structure of the initial growth state of carbon “fiber seeds” located on the surfaces of the nanoporous multicrystal copper catalyst particles. The difference in the contrast suggests that the carbon source gas presents selectivity for different catalytically active crystal planes of the nanoporous multicrystal copper catalyst particles during the adsorption process, among which, the (111) plane shows high catalytic activity.^[^ ^16^ ^]^ Moreover, the high‐resolution transmission electron microscopy (TEM) (HRTEM) image of the nanoporous multicrystal copper particles in Figure S4 (Supporting Information) further verifies that dense crystal planes are uniformly distributed on their surfaces. The carbon “fiber seeds” have longitudinal and intertwined structures, which become the framework of the BPCFs with the increase in the growth time (Figure S5, Supporting Information). This process creates a porous structure and introduces abundant vacancy defects during the growth of the CNFs. In addition, we find that the basic frame of the BPCFs is formed after 30 min of growth, and BPCFs with an average diameter of 300 nm are obtained after 60 min, as shown in Figure 2c,d, respectively. A magnified SEM image of the BPCFs in Figure S6 (Supporting Information) shows that the above‐mentioned porous structure is formed on the surfaces and in the interior of the CNFs, further confirming the characteristics of an interwoven growth (Figure S6, Supporting Information). This 3D porous network structure can increase the specific surface area, providing active sites for Na^+^. Simultaneously, the 3D porous network can efficiently reduce the transport distance of ions/electrons and promote the buffering capacity during the sodiation/desodiation process. Uniform BPCFs with lengths of several micrometres are interconnected, which can be beneficial for electron conductivity (Figure S6b, Supporting Information). Figure 2e shows that a thin layer of amorphous carbon is formed on the surfaces of the BPCFs, which can produce many defects, which provide numerous adsorption sites for Na^+^. The HRTEM images of BPCFs shown in Figure 2f with different contrasts further demonstrate the presence of abundant nanoscale porous structures on the surface and in the interior of the BPCFs. These 3D interconnected porous networks can be used as reservoirs for the electrolyte, contributing to efficient Na^+^ transport. The above‐mentioned structural characteristics are consistent with the SEM characterization results. Furthermore, the formed porous structures can induce disorder in the CNFs. The HRTEM images also suggest that the BPCFs mainly contain randomly amorphous carbon and partially crystalline carbon, as also shown in the SAED pattern (upper right inset in Figure 2g). The spacing between the interlayers is ≈0.387 nm, which can provide large intercalation spaces to accommodate Na^+^ and reduce the kinetic energy consumption. Moreover, the diameter of the BPCFs having a homogeneous texture is consistent with that of the NPC, suggesting that the catalyst particles affect not only the microstructure but also the macroscopic characteristics of the carbon product (Figure S7, Supporting Information). Concurrently, the color of the surface of the catalytic substrate changes from metallic lustre to carbon black, suggesting the formation of a carbon product after addition of the carbon source gas for 60 min at 600 ℃ (Figure S8, Supporting Information). The yield of the deposited carbon reaches a maximum of 140% with a growth time of 60 min (Figure S9, Supporting Information).

**Figure 3:**
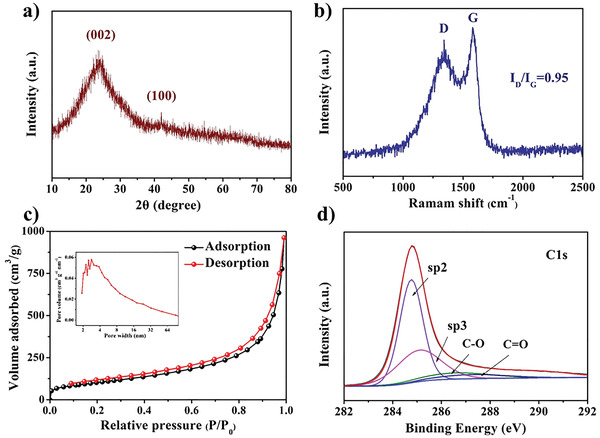
a) XRD pattern and b) Raman spectrum of BPCFs. c) Nitrogen adsorption–desorption isotherms of BPCFs (inset shows pore size distribution). d) High‐resolution XPS C1s spectrum of BPCFs.

**Figure** **3**a shows the X‐ray diffraction (XRD) pattern of the as‐prepared BPCFs. Two remarkable broad diffraction peaks are observable, which correspond to the (002) and (100) planes of graphite, respectively.^[^ ^19^ ^]^ Their broadness suggests the amorphous properties of the BPCFs.^[^ ^20^ ^]^ The Raman spectrum of the BPCFs is shown in Figure 3b. Two distinct characteristic peaks are observed at ≈1340 and ≈1580 cm^−1^, which are associated with the D and G bands of graphite, respectively. The D band indicates the defects and disorder degree of carbon, and the G band is the result of *sp* ^2^ stretching. The quality of carbon can be determined by comparing the *I*_D_/*I*_G_ ratio. For the BPCFs, the *I*_D_/*I*_G_ ratio is ≈0.95, suggesting poor crystallinity.^[^ ^21^ ^]^ This characteristic can provide abundant vacancy defects for the adsorption of Na^+^.^[^ ^22^ ^]^

Figure 3c shows the typical N_2_ adsorption–desorption isotherms and pore size distribution of the BPCFs. The isotherms from the Brunauer–Emmett–Teller (BET) analysis are typical type‐IV curves with a hysteresis loop in the readsorption branch, suggesting that the BPCFs contain numerous mesopores.^[^ ^23^ ^]^ The BPCFs offer a large specific surface area of ≈372 m^2^ g^−1^, indicating that the sample can provide sufficient number of active sites for Na^+^. Furthermore, the pore size distribution of the BPCFs is principally in the range of ≈2–5 nm, and a large part of the mesopores can offer rapid transmission channels for the electrolyte and ions/electrons. To further explain the characteristics of the BPCFs, we obtained the data of X‐ray photoelectron spectroscopy (XPS). As shown in Figure S10 (Supporting Information), there are two distinct peaks at ≈285 and 532 eV corresponding to the C1s and O1s peaks, respectively, suggesting that the BPCFs contain both C and O elements. Fitting of the C1s peak yields four energy peaks located at 284.76, 285.1, 286.6, and 287.3 eV, corresponding to C‐sp^2^, C‐sp^3^, C—O, and C═O, as shown in Figure 3d. The appearance of the C‐sp^3^ peak indicates the presence of defects in the sample.^[^ ^24^ ^]^ The high percentage of C‐sp^3^ supports the formation of numerous vacancy defects (Table S2, Supporting Information). The high‐resolution XPS O1s spectrum in Figure S11 (Supporting Information) shows two binding energy peaks at 530.9 and 532.4 eV, corresponding to C═O and C—O bonds. The entire preparation of BPCFs does not involve oxygen or oxygen‐containing compounds. Therefore, the small number of oxygen‐containing groups probably originates from oxygen adsorption during the transfer of the sample, which could react with sodium to form some irreversible compounds (a solid electrolyte interphase (SEI)) during the initial discharge process.

**Figure 4:**
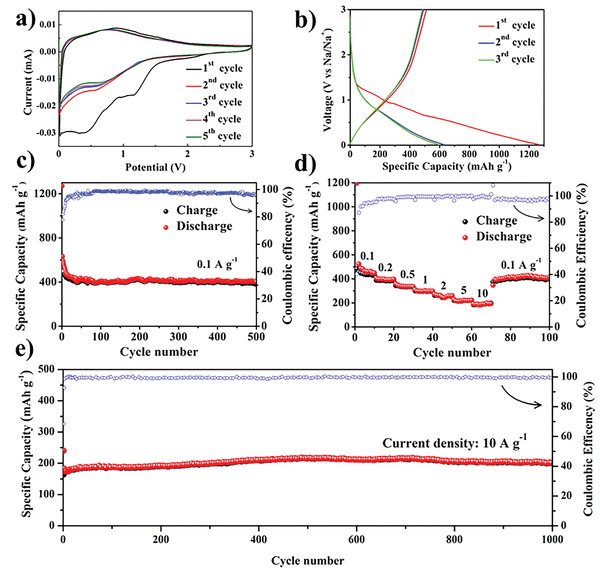
a) CV curves (scan rate: 0.1 mV s^−1^) and b) discharge/charge voltage profiles (current density: 0.1 A g^−1^) of BPCFs electrode. c) Cycling performance at 0.1 A g^−1^ and d) rate capability of BPCFs electrode. e) Long‐term cycling performance of BPCFs electrode at 10 A g^−1^ with a high‐mass‐loading of 5 mg cm^−2^.

The electrochemical performance of the BPCFs was evaluated by cyclic voltammetry (CV), galvanostatic charging–discharging, and electrochemical impedance spectroscopy (EIS). **Figure** **4**a shows the CV curves of the BPCFs obtained with a scan rate of 0.1 mv s^−1^. In the first discharge cycle, the distinct irreversible peaks at 1.17 V can be assigned to the formation of an SEI film, which can cause partial sodium ion consumption and electrolyte degradation.^[^ ^25^ ^]^ The reduction peak at 0.4 V is attributed to the insertion of Na^+^ into the electrode material.^[^ ^26^ ^]^ In the following cycles, the curves almost overlap, suggesting the formation of a stable SEI film. Figure 4b presents the first three discharge/charge voltage profiles of the BPCFs at a current density of 0.1 A g^−1^. In the first discharge curve, discharge voltage plateaus from 1.3 to 0.9 V and from 0.7 to 0.3 V are observed, which correspond to the irreversible peaks in the CV curve. Furthermore, in the following cycles, the degrees of overlap in the discharge/charge voltage profiles and the CV curves are similar, which shows that the BPCF electrode has excellent reversibility. The reversible discharge capacity of the BPCFs as an electrode material at a current density of 0.1 A g^−1^ maintains excellent cyclic stability during the charging and discharging cycles (Figure 4c). The Coulombic efficiency approaches 99% after 100 cycles. The specific capacitance of the BPCFs retains its high discharge capacity of 400 mAh g^−1^ after 500 cycles, which does not undergo significant capacitance attenuation. Correspondingly, the microstructure of the BPCFs does not remarkably change after 500 cycles (Figure S12, Supporting Information), which further verifies the excellence of the braided porous structure. The BPCFs show remarkable sodium storage ability and stable cycling performance compared with other advantageous carbon material electrodes (Table S3, Supporting Information). Concurrently, control samples of straight‐walled carbon fibers (SWCFs) were prepared under similar experimental conditions as the BPCFs. To prevent the recrystallization and coarsening of the NPC during heating, the copper catalyst and the carbon source gas, i.e., acetylene, were directly sent to a heating zone preheated to 600 ℃. From Figure S13 (Supporting Information), the diameter of the SWCFs is ≈50 nm, which is smaller than that of the BPCFs (200 nm, Figure 2). Moreover, the SWCFs do not possess a porous structure owing to the lack of the catalytically active (111) planes. The SWCFs present electrochemical properties similar to those of the BPCFs. However, the capacity of the former is approximately half of that of the latter (430 mAh g^−1^). Specifically, their capacity is 192 mAh g^−1^ after 200 cycles at a current density of 0.1 mA g^−1^, as presented in Figure S14 (Supporting Information). The high specific capacitance may be attributed to the vacancies providing sufficient adsorption sites for Na^+^, and the outstanding cyclic stability can be attributed to the stable framework of the 3D interconnected networks.^[^ ^27^ ^]^ The rate performance of the active electrode material was further analyzed by changing the current density. The results in Figure 4d show that the BPCFs anode achieves reversible discharge capacities of 449, 397, 335, 298, 260, 223, and 195 mAh g^−1^ at 0.1, 0.2, 0.5, 1, 2, 5, and 10 A g^−1^, respectively. More promisingly, the BPCFs still show a high discharge capacity of 411 mAh g^−1^ when the current density is recovered to 0.1 A g^−1^. The rate performance of the BPCFs shows a high discharge capacity among recently reported carbon materials (Figure S15, Supporting Information). As shown in Figure 4e, the long‐term performance is tested using a high‐mass‐loading BPCFs electrode with 5 mg cm^−2^ at a high current density of 10 A g^−1^. The initial Coulombic efficiency (68.4%) at a high current density (10 A g^−1^) is significantly higher than that measured at 0.1 A g^−1^ (40.35%). This is because the electrochemical reaction rate cannot match with the voltage drop; therefore, more sodium ions are mainly inserted in the nanopores and the crystalline carbon layer with a large layer spacing in the BPCFs. The initial Coulombic efficiency of carbon‐based materials can be effectively improved using an appropriate electrolyte. We configured 1 m NaPF_6_/dimethoxyethane (DME) and 1 m NaPF_6_/tetraethylene glycol dimethyl ether (TEGDME) (Figure S16, Supporting Information) to improve the initial Coulombic efficiency. Figure S16 (Supporting Information) shows that the initial Coulombic efficiency of a coin cell with NaPF_6_/DME is up to 78.61%, whereas those of the coin cells with NaClO_4_/ethylene carbonate (EC)/diethyl carbonate (DEC)/fluoroethylene carbonate (FEC) and NaPF_6_/TEGDME are 40.35% and 20.29%, respectively. The discharge capacity of the BPCFs shows a gradual increase in the first 500 cycles, indicating that a new irreversible SEI film gradually forms on their surfaces as the cycle progresses at 10 A g^−1^ (Figure 4e). Subsequently, the discharge capacitance is almost maintained at 201 mAh g^−1^ after 1000 cycles with a Coulombic efficiency of 99.9%, suggesting remarkable cycling stability for high‐power SIBs. The BPCFs electrode with mass loading of 1 mg cm^–2^ show a similar cycling performance at a current density of 10 A g^−1^ after 5000 cycles (Figure S17, Supporting Information). To verify the Coulombic efficiency of the CNFs in a practical battery, we assembled BPCFs||BPCFs symmetric cells (Figures S18 and S19, Supporting Information) and BPCFs||Na_3_V_2_(PO_4_)_3_ full cells (Figure S20, Supporting Information), in which all electrodes were activated before use in the cells. The Coulombic efficiency of the BPCFs||BPCFs symmetric cells was maintained at 97.6% after ten cycles (Figure S19, Supporting Information). The BPCFs||Na_3_V_2_(PO_4_)_3_ full cells also showed excellent Coulombic efficiency. After ten cycles, their Coulombic efficiency stabilized at ≈99% from the initial 65.96% (Figure S20, Supporting Information). Moreover, for an in‐depth understanding of the cycling performance of the BPCFs, we prepared electrodes with different active material mass loadings: 2, 3, and 5 mg cm^−2^. As presented in Figure S21 (Supporting Information), with the increase in the mass loading, the specific capacity and cycle performance of the BPCFs electrode show good stability. The 5 mg cm^−2^ electrode shows a capacity of 295 mAh g^−1^ after 100 cycles with a current density of 0.1 mA g^−1^, whereas the capacities of the 2 and 3 mg cm^−2^ electrodes increase up to 370 and 335 mAh g^−1^, respectively, under the same test conditions. The excellent fast charge and discharge capability of the BPCFs electrode may be ascribed to the interconnected porous structure, which shortens the diffusion distance of Na^+^ and electrons, and the large interlayer distance (0.387 nm), which provides sufficient transmission space. Concurrently, the 3D porous structure provides a framework support for long cycles.

To gain further insight into the structure of the electrochemical systems and the properties of the electrode processes, EIS measurements were conducted on the BPCF electrode. As shown in Figure S22 (Supporting Information), the Nyquist plot consists of a sloping straight line in the low‐frequency region and a semicircle in the high‐frequency region, which are controlled by diffusion and charge transfer, respectively.^[^ ^28^ ^]^ The corresponding fitted equivalent circuit model is shown in the inset of Figure S22 (Supporting Information). The charge transfer resistance (*R*_ct_) of the BPCFs is 190 Ω, which is superior to those of previously reported porous carbon‐based anodes (Table S4, Supporting Information). These results suggest a fast kinetic reaction of the BPCFs electrodes, which further confirms the structural excellence of the BPCFs in the field of sodium storage.

**Figure 5:**
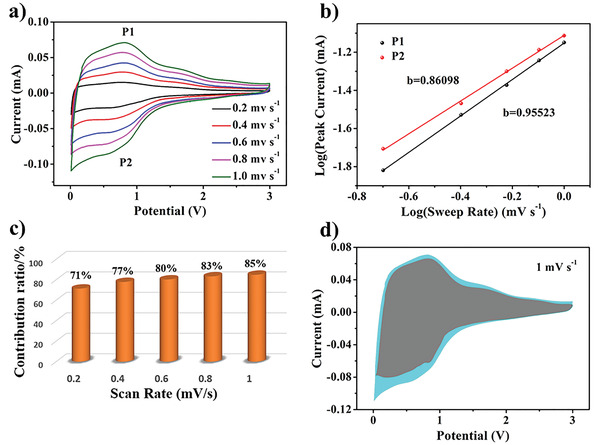
Na^+^ storage kinetics and quantitative analysis of BPCFs. a) CV curves at various scan rates ranging from 0.2 to 1.0 mV s^−1^. b) Log(i) versus log(v) plots of cathodic and anodic peaks. c) Normalized contribution of capacitive‐controlled charge versus scan rate of BPCFs. d) Capacitive (origin blue region) and diffusion‐controlled contributions to charge storage at 1 mV s^−1^.

For a better analysis of the remarkable electrochemical performance, the electrochemical kinetics of the BPCFs electrode was explored by CV measurements. **Figure** **5**a presents the CV curves at scan rates from 0.2 to 1.0 mv s^−1^ for determining the charge storage mechanism. With continuously increasing scan rates, the counterpart CV curves present similar shapes and visible peaks. Accordingly, the peak current (*i*) and the scan rate (*v*) are related by the following rigorous equation

(1) $$i=av^{b}$$

where *a* and *b* are correlation parameters. The major capacity contributions originate from the diffusion process and the pseudocapacitive behavior.^[^ ^29^ ^]^ The storage of sodium ions correlates with the pseudocapacitive behavior when *b* is ≈1, whereas *b* = 0.5 implies that the diffusion‐controlled behavior determines the capacity. Consequently, Figure 5b shows the CV curves with *b* values 0.95 523 and 0.86 098, corresponding to the P1 and P2 peaks, respectively. The curves reveal that the dynamics of the BPCFs are controlled by the capacitive behavior, indicating that Na^+^ adsorption on the surfaces of the CNFs is the main charge storage mechanism. A quantitative analysis was performed to further explore the relationship between the sweep rate (*v*) and the effect of the pseudocapacitive behavior on the charge storage. The contribution rate is calculated using the following equation

(2) $$i\left(v\right)=k_{1}v+k_{2}v^{1/2}$$

where *i*(*v*) represents the current of the CV curve at a certain voltage. *v* denotes different scan rates. *k*_1_ and *k*_2_ are fitting parameters. Figure 5c shows the results of the quantitative analysis, based on which the capacitance contribution rate is enhanced with the increase in the sweep rate from 0.2 to 1.0 mv s^−1^. In particular, the pseudocapacitive contribution rate reaches up to 85% when the scan rate is 1.0 mv s^−1^ (Figure 5d). This result reasonably explains the excellent performance rate of the BPCFs with porous structures, even at high current densities.

**Figure 6:**
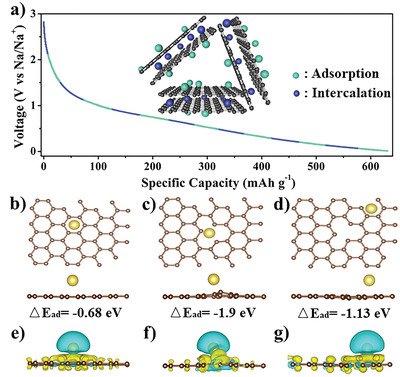
a) Discharge voltage profiles (current density: 0.1 A g^−1^) of BPCFs and schematic of proposed sodium storage mechanism. Stack thickness and plane size are not to scale, and defects and curvature are not explicitly shown. Simulation and adsorption site of Na atom on b) ideal carbon structures, c) single vacancy, and d) carbon layer edge. e–g) Side views of difference charge density of Na^+^ absorbed by different carbon structures. Yellow and blue regions represent charge accumulation and depletion, respectively. Brown and yellow balls represent C and Na atoms, respectively.

To further understand the high‐efficiency sodium storage mechanism of the BPCFs, density functional theory (DFT) calculations were conducted to explore the effects of vacancies on the Na^+^ adsorption behavior. The discharge voltage curves of the BPCFs with abundant vacancies and a large interlayer space (0.387 nm) were combined to study with the sodium storage mechanism (**Figure** **6**a). According to conventional studies, the existence of a large proportion of a sloping region in the discharge voltage curve suggests that the sodium storage capacity can be attributed to the absorption and intercalation of Na^+^.^[^ ^30^ ^]^ In the chosen BPCFs, the large interlayer spacing (0.387 nm) is available for Na^+^ transmission and intercalation (Figure 2).^[^ ^31^ ^]^ Considering the adsorption sodium storage mechanism, the sites with defects in the carbon materials have the highest adsorption energy for sodium ions.^[^ ^30^ ^]^ However, the adsorption capacity of Na^+^ at the edges around defects has not been systematically discussed. Concurrently, the BPCFs possess disordered graphite structures with many edges (Figure 2g). Correspondingly, the adsorption energies of Na^+^ on vacancies and edges were calculated via first‐principles calculations, to explore the sodium storage mechanism of the BPCFs (Figure 6; and Figures S23 and S24, Supporting Information). Figure 6b–d; and Figures S23 and S24 (Supporting Information) show the typical models of Na^+^ on ideal, mono‐vacancy, and three‐edge position carbon layers, respectively. The DFT calculation results prove that the vacancy on the carbon surface not only significantly improves its storage capacity of Na^+^ but also increases its electronic conductivity. ^[^ ^32^ ^]^ Compared to the adsorption energy (△*E*_ad_) (−0.68 eV) of Na^+^ on the ideal carbon layer, the △*E*_ad_ of Na^+^ on the mono‐vacancy carbon layer is −1.9 eV, which suggests that Na^+^ is more stable on the mono‐vacancy carbon layer than on the ideal carbon layer. This result shows that the mono‐vacancy increases the Na^+^ adsorption ability. Interestingly, the calculated results show that the defect edges also have high Na^+^ adsorption ability, and the adsorption energy decreases with the increase in the distance from the vacancy (−1.28, −1.13, and −1.09 eV, respectively). This feature clarifies that the entire carbon layer with vacancies has a high Na^+^ adsorption energy, and not only the vacancy position. Therefore, based on the characteristics of the slope discharge curve, the sodium storage mechanism of the BPCFs includes both adsorption and intercalation, and the adsorption mechanism is dominant because of its highly disordered graphite structure. The 3D charge density difference further reveals the effect of the vacancy for the adsorption of Na^+^ from the perspective of charge. It can be observed that the charge density is reconstructed after Na^+^ absorption (Figure 6e–g; and Figures S23 and S24, Supporting Information), which shows a charge transfer from Na^+^ to its nearest C atom. Particularly, compared to the uniformly distributed charge density on the entire structure when Na^+^ is adsorbed on the ideal carbon layer, the charge tends to accumulate around the vacancy and the edges with a low charge density. Therefore, high‐density vacancies can efficiently improve the sodium storage efficiency of these CNFs.

## 3 Conclusion

In summary, BPCFs with a stable framework were prepared via a one‐step in situ catalytic synthesis method using a nanoporous multicrystal copper as the catalyst. Carbon “fiber seeds” were initially grown on high‐catalytic activity crystal planes of the catalyst particles. The adjacent carbon “fiber seeds” were interwoven along the direction of the fiber length, becoming the framework of the BPCFs and introducing abundant vacancies. The BPCFs, having a stable frame and porous network structures, present remarkable electrochemical performance with a high charge/discharge capacity of 401 mAh g^−1^ at 0.1 A g^−1^ after 500 cycles. The calculated results confirm that the considered vacancy and its near edges could effectively enhance the Na + adsorption capacity. Therefore, these results lead to broadening of the applications of SIBs in large‐scale energy storage systems.

## 4 Experimental Section

### Preparation of 3D NPC

Cu_30_Mn_70_ alloy strips with a thickness of 30 µm were prepared using smelting belt swing technology. Mn in Cu_30_Mn_70_ was corroded out using the dealloying method, in aqueous HCl solution (0.025 mol L^−1^) at 40 ℃ in a vacuum chamber, and the interconnected 3D NPC was obtained.

### Synthesis of BPCFs Structure

The as‐prepared 3D NPC was first placed in a furnace and immersed in a mixed gas of acetylene (C_2_H_2_) (10 sccm) and Ar (250 sccm) at 600 ℃ for 1 h and cooled to 20 ℃ in an Ar‐protecting atmosphere. BPCFs were uniformly grown on the surface of the 3D NPC. Subsequently, the 3D NPC with BPCFs was corroded by immersion in FeCl_3_ (5 g) + HCl (10 mL) + H_2_O (100 mL) solution for 12 h. Subsequently, it was dipped in concentrated nitric acid solution (68% purity) for 3 h at room temperature. Finally, the BPCFs were rinsed with deionized water and vacuum‐dried.

### Materials Characterization

The microstructures and morphologies of an as‐prepared sample were investigated by field emission SEM (FE‐SEM, S‐8100, Hitachi) and TEM (FEI Tecnai G2 S‐Twin instrument with a field emission gun operating at 200 kV). The structure of the carbon fibres was characterized by powder XRD (Rigaku D/Max‐2400 with Cu K*α* radiation). The graphitization structure was verified using a Raman spectrometer (X ploRA PLUS; laser: 632.8 nm). The specific surface areas and pore size distributions of as‐prepared samples were measured using the N_2_ adsorption–desorption method (BET, Autosorb‐iQ‐C from Quantachrome). The XPS spectra were collected with an ESCALAB 250 X‐ray photoelectron spectrometer with an Al Ka X‐ray source (*hν* = 1486.6 eV).

### Electrochemical Characterizations

The electrochemical performance was tested in 2032‐type coin cells. An active electrode was prepared by uniformly mixing the active material (80 wt%), a conductive agent (carbon black) (10 wt%), and a binder (polyvinylidene fluoride) (10 wt%) in *N*‐methyl‐pyrrolidone, and coated on a Cu foil. Subsequently, the sample was dried in a vacuum chamber at 60 ℃ for 24 h. Finally, 2032‐type coin cells were assembled in an Ar‐filled glove box (H_2_O and O_2_ < 0.1 ppm; MBRAUN). The mass loadings of the active material were ≈1, 2, 3, and 5 mg cm^−2^, respectively. The fibreglass diaphragm caused a separation effect. Sodium pieces as the counter electrode provided a large number of Na^+^. As an electrolyte, 1 m NaClO_4._ in an EC/diethyl carbonate (1:1, vol ratio) solution with 5% FEC additive was used. Note that 1 m NaPF_6_ in DME and 1 m NaPF_6_ in TEGDME were used as comparison electrolytes. CV and EIS) (frequency range: 0.01 Hz–100 kHz) were measured on a Biologic electrochemical workstation (Versatility VMP‐300, France). Galvanostatic charge/discharge and cycle stability tests were conducted using a LAND CT2001A multichannel battery testing system (vs Na^+^/Na).

### DFT Calculations

All DFT calculations were performed using the Vienna ab initio simulation package. The exchange–correlation functional was described using the Perdew–Burke–Ernzerhof generalized gradient approximation method. All self‐consistent calculations were performed with a plane‐wave cut‐off of 500 eV. The convergence accuracy of the self‐consistent process was set as 10^−6^ eV, and the geometrical optimization was stopped when the Hellmann–Feynman forces on the atoms were smaller than 0.02 eV Å^−1^. Brillouin zone k‐point sampling was performed with 3×3×2 Γ‐centered Monkhorst–Pack grids. A vacuum space of 20 Å was constructed to eliminate the interactions between adjacent layers. The adsorption energy (*E*_ad_) was calculated using the following equation

(3) $$E_{\mathrm{ad}}=E_{c+\mathrm{na}}-E_{c}-E_{\mathrm{na}}$$

where *E*_c+na_ and *E*_c_ are the total energies of the system before and after sodium absorption, respectively, and *E*_na_ is the energy of a sodium atom. A positive *E*_ad_ indicates that intercalation or storage is energetically unfavorable.

## Conflict of Interest

The authors declare no conflict of interest.

## Supporting information

Supporting Information

## Data Availability

The data that support the findings of this study are available from the corresponding author upon reasonable request.
