# Supplementary material for: Architecting Braided Porous Carbon Fibers Based on High‐Density Catalytic Crystal Planes to Achieve Highly Reversible Sodium‐Ion Storage
Source: Adv Sci (Weinh). 2022 Apr 26;9(18):2104780. doi: 10.1002/advs.202104780 (PMC9218750; doi:10.1002/advs.202104780)
Supplement: Supplementary file 1 — Supporting Information [file ADVS-9-2104780-s001.pdf]

ADVANCED SCIENCE
Open Access

Supporting Information

for *Adv. Sci.*, DOI 10.1002/advs.202104780

Architecting Braided Porous Carbon Fibers Based on High-Density Catalytic Crystal Planes to Achieve Highly Reversible Sodium-Ion Storage

Chuanqi Li, Zhijia Zhang, Yuefang Chen, Xiaoguang Xu, Mengmeng Zhang, Jianli Kang, Rui Liang, Guoxin Chen, Huanming Lu, Zhenyang Yu, Wei-Jie Li, Nan Wang, Qin Huang, Delin Zhang, Shu-Lei Chou* and Yong Jiang**

Supporting Information

Architecting braided porous carbon fibres based on high-density catalytic crystal planes to achieve highly reversible sodium-ion storage

Chuanqi Li[1#], *Zhijia Zhang*[1#]*, *Yuefang Chen*[1], *Xiaoguang Xu*[2], *Mengmeng Zhang*[1], *Jianli Kang*[3], *Rui Liang*[4], *Guoxin Chen*[4], *Huanming Lu*[4], *Zhenyang Yu*[1], *Wei-Jie Li*[5], *Nan Wang*[6], *Qin Huang*[7], *Delin Zhang*[1], *Shu-Lei Chou*[8]*, *Yong Jiang*[1]*

[1]State Key Laboratory of Separation Membrane and Membrane Processes, Tianjin Municipal Key Laboratory of Advanced fibres and Energy Storage, School of Materials Science and Engineering, School of Electronic and Information Engineering, School of Mechanical Engineering, Tiangong University, Tianjin 300387, China

[2]School of Materials Science and Engineering, University of Science and Technology Beijing, Beijing 100083, China

[3]School of Materials Science and Engineering, Tianjin University, Tianjin 300072, China

[4]Ningbo Institute of Materials Technology & Engineering, Chinese Academy of Sciences, Ningbo 315201, China

[5]Institute for Superconducting and Electronic Materials, University of Wollongong, Wollongong, NSW 2522, Australia

[6]China Electronics Technology Group Corporation No. 46 Institute, CETC JH(Tianjin) Semiconductor Material Co. Ltd., Tianjin, 300220, China

[7]Guangdong Institute of Semiconductor Industrial Technology, Guangdong Academy of Sciences, Guangzhou 510651, China

[8]Institute for Carbon Neutralization, College of Chemistry and Materials Engineering，Wenzhou University, Wenzhou, Zhejiang 325035, China

---

[#] Chuanqi Li and Zhijia Zhang contributed equally

*Corresponding Author: A/Prof. Zhijia Zhang, E-mail address: zhangzhijia@tiangong.edu.cn

Prof. Shulei Chou, E-mail address: chou@wzu.edu.cn

Prof. Yong Jiang, E-mail address: yjiang@tiangong.edu.cn

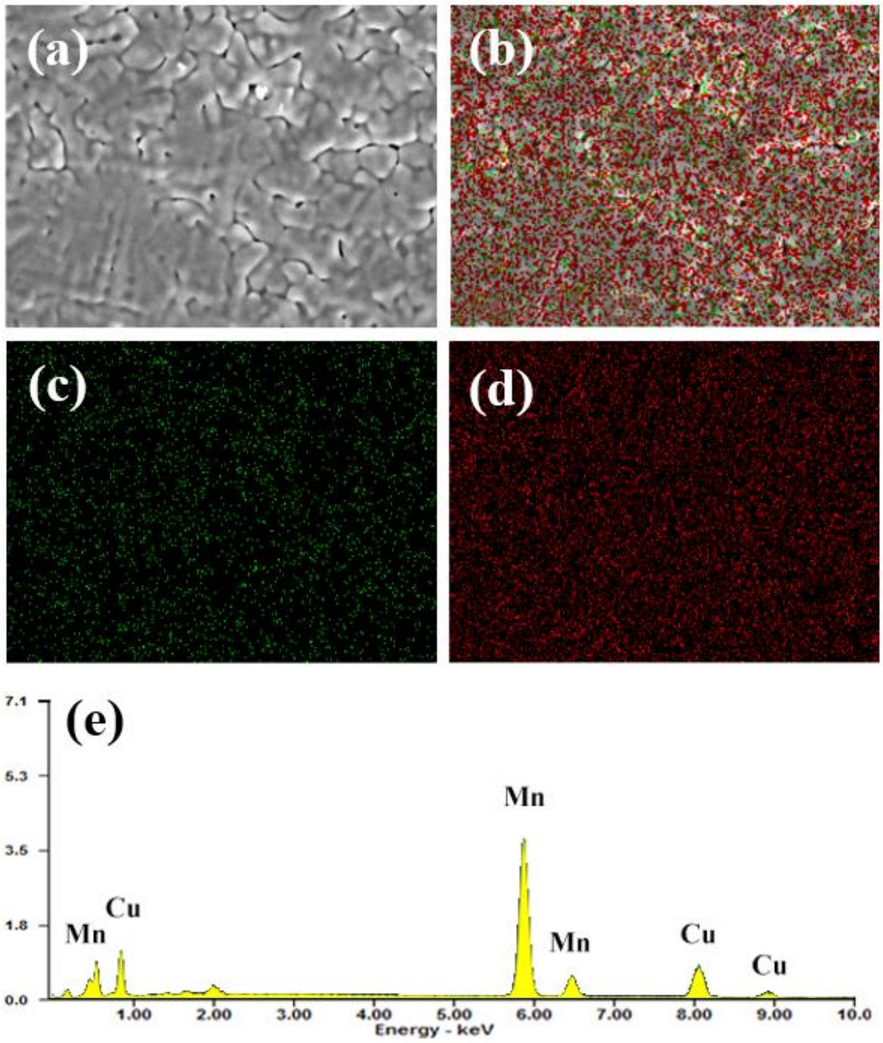

Figure S1. (a) The scanning electron microscope (SEM) image of $Cu_{30}Mn_{70}$ alloy. (b) The energy dispersive X-ray spectroscopy (EDS) layered image of $Cu_{30}Mn_{70}$ alloy and corresponding (c) Cu, (d) Mn elemental mappings. (e) Energy Dispersive X-Ray Spectroscopy (EDX) analysis of $Cu_{30}Mn_{70}$ alloy.

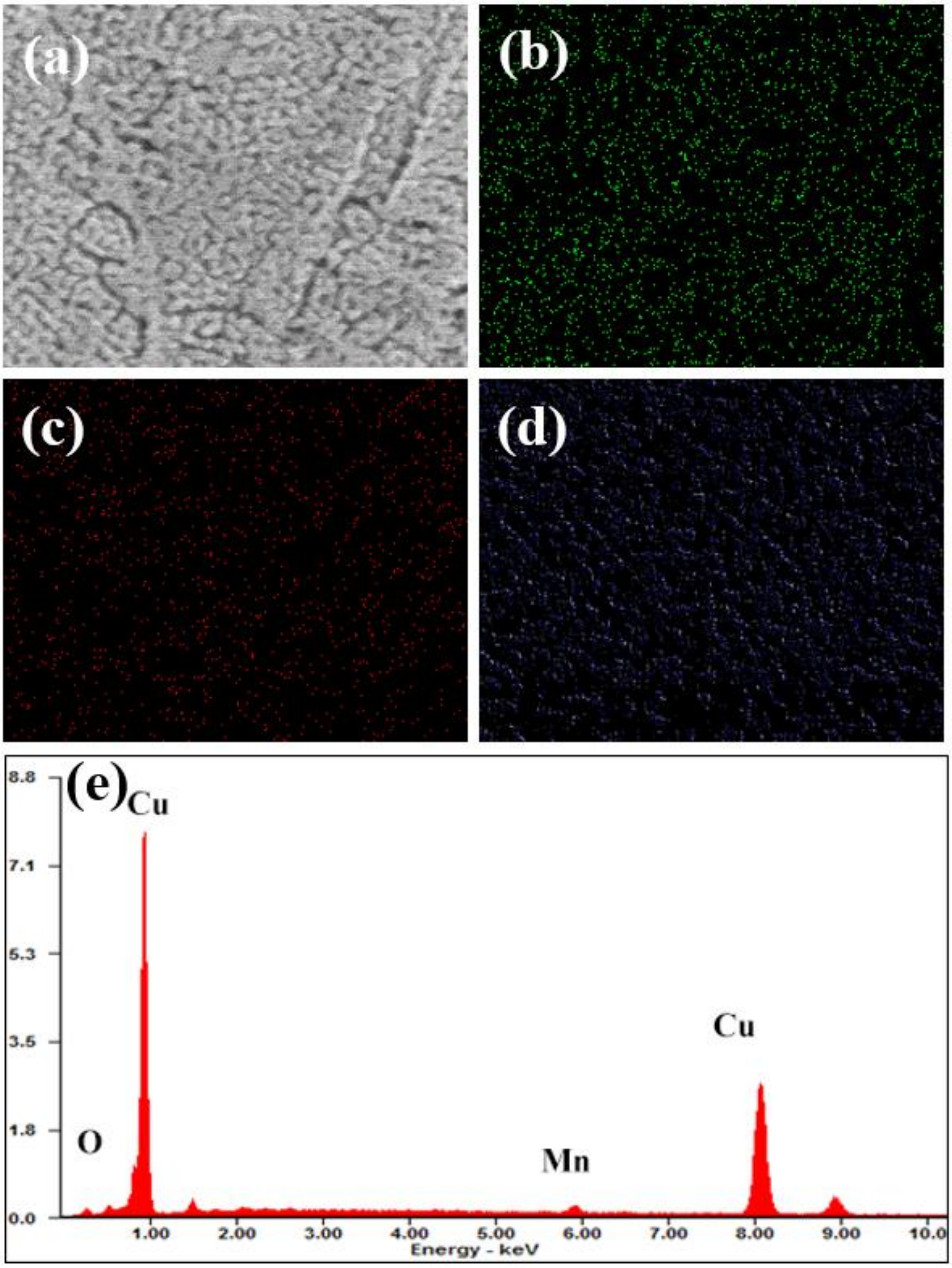

Figure S2. (a) SEM images of 3D NPC, and corresponding (b) Cu, (c) Mn, and (d) O elemental mapping images. (e) EDX analysis of 3D NPC.

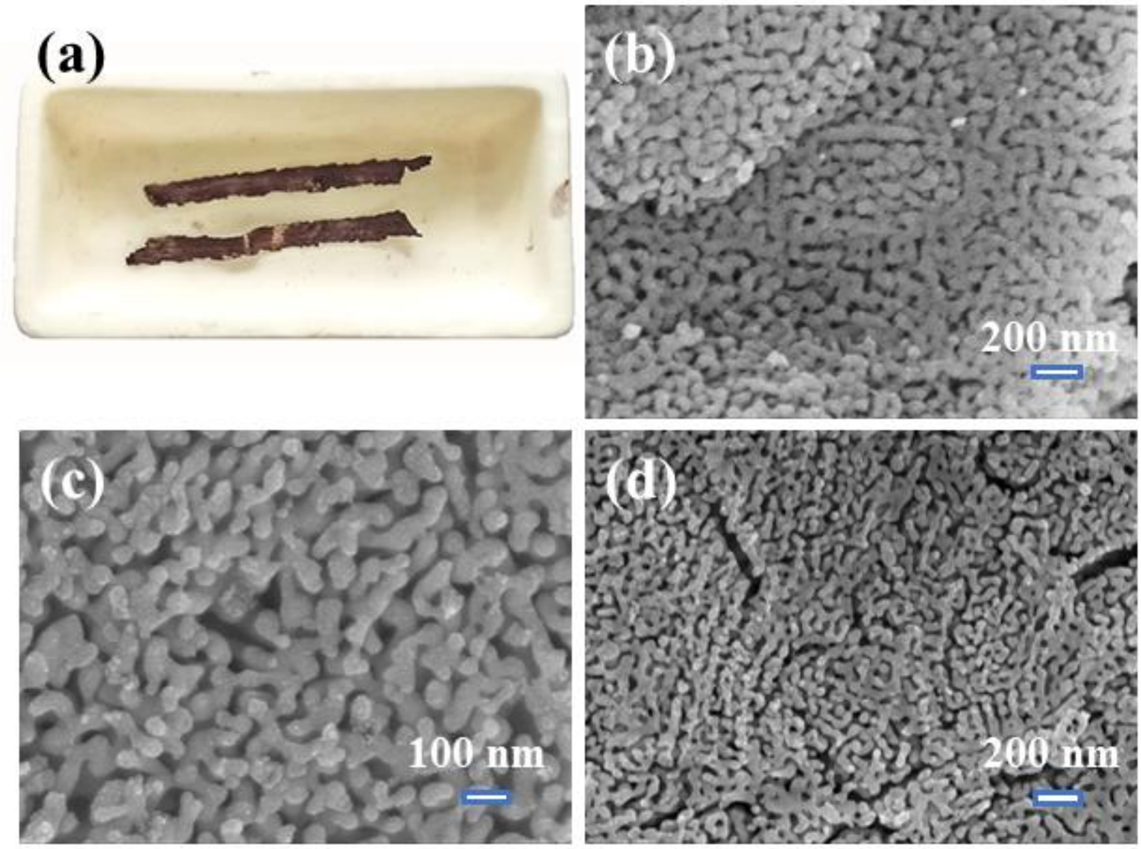

Figure S3. (a) Photograph of 3D NPC in the crucible. SEM images of 3D NPC (b) in the cross-section, (c and d) in the front.

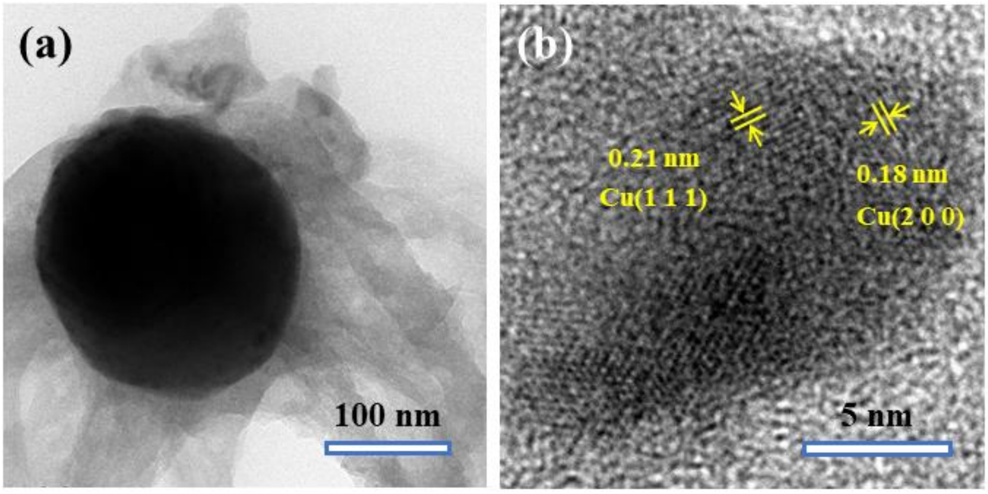

Figure S4. (a) TEM image, (b) high-resolution TEM analysis of nanoporous multi-crystal copper catalyst particle after grown for 30 min.

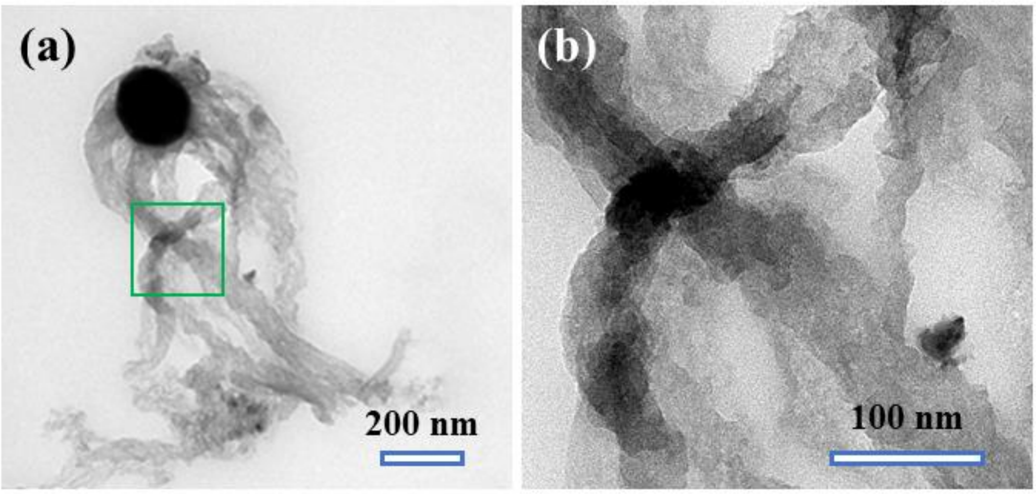

Figure S5. (a) TEM image, (b) HRTEM image of carbon “fiber seeds” after grown for 30 min.

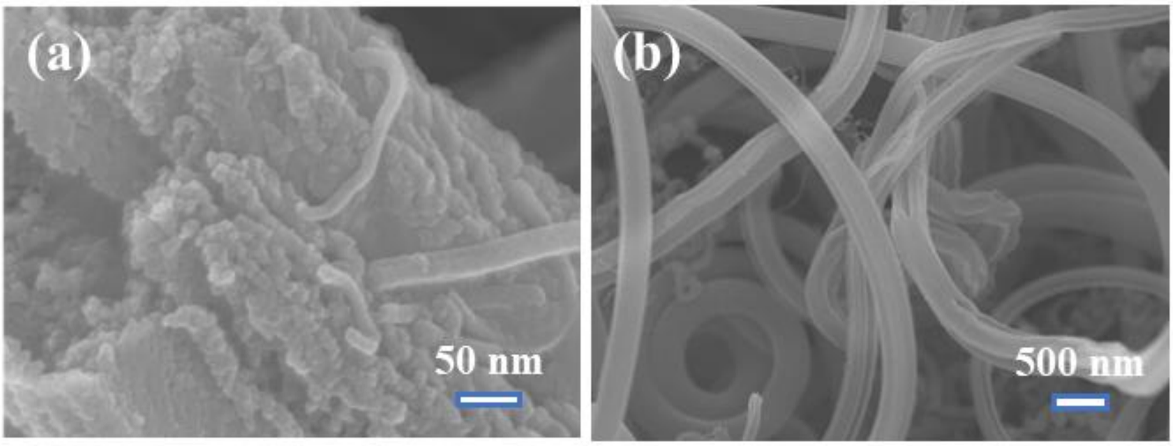

Figure S6. SEM images of BPCFs after grown for 60 min.

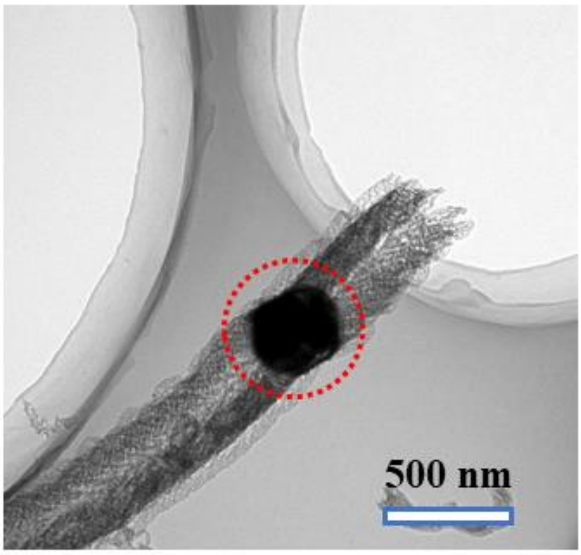

Figure S7. TEM images of BPCFs after growing for 60 min.

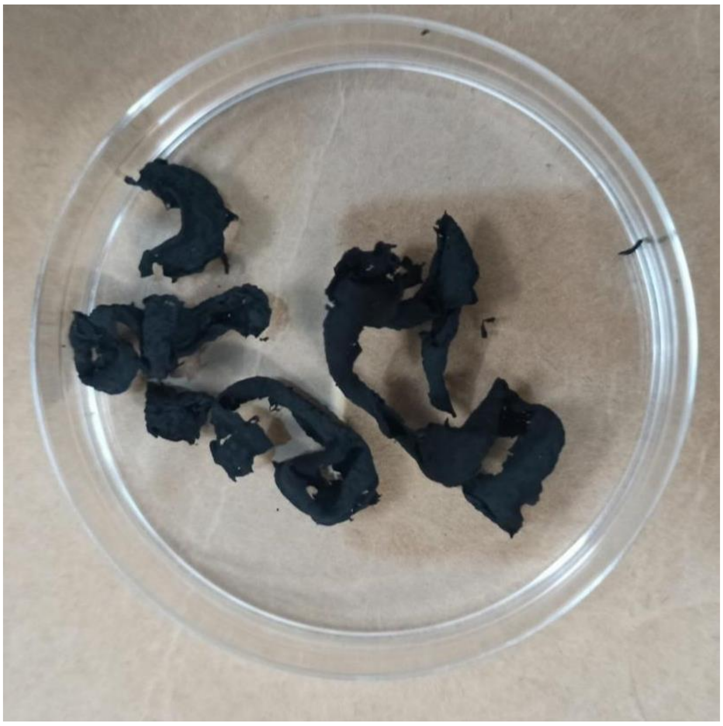

Figure S8. The picture of BPCFs growing for 60 minutes.

The yield of deposited carbon can be qualitatively determined by the following equation:

$$C\ (\text{wt.\%}) = \frac{M_1 - M_2}{M_2} * 100\% \qquad (1)$$

with M_1 representing the quality of the product after the reaction; M_2 corresponding to the quality of the catalyst.

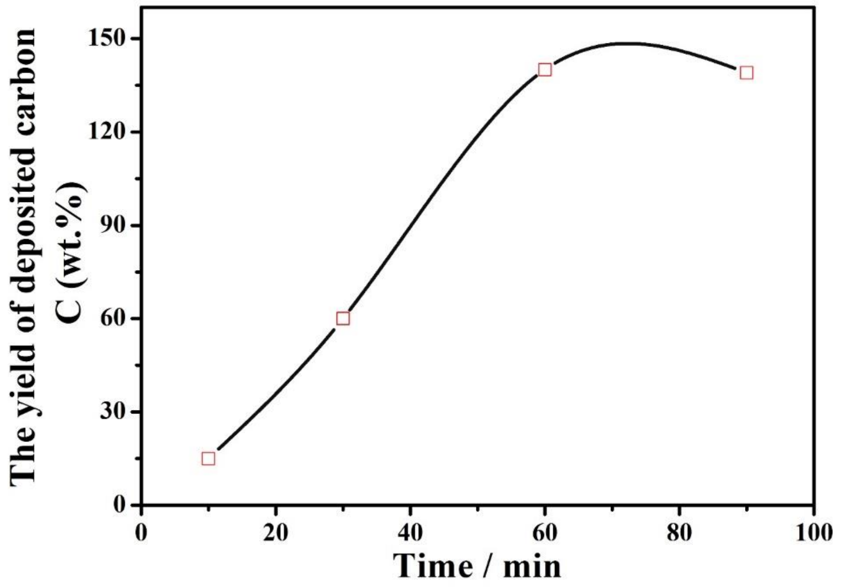

Figure S9. Carbon yield curve with the different synthesis times.

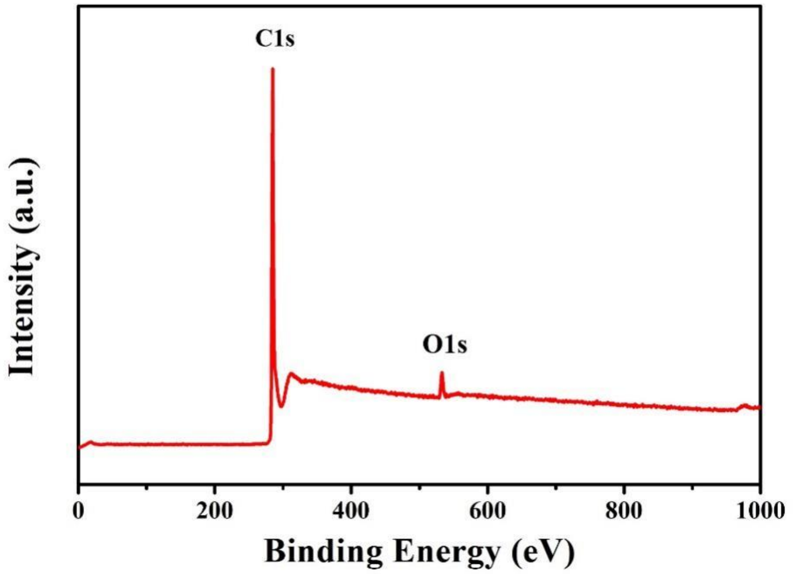

Figure S10. XPS survey spectrum of BPCFs.

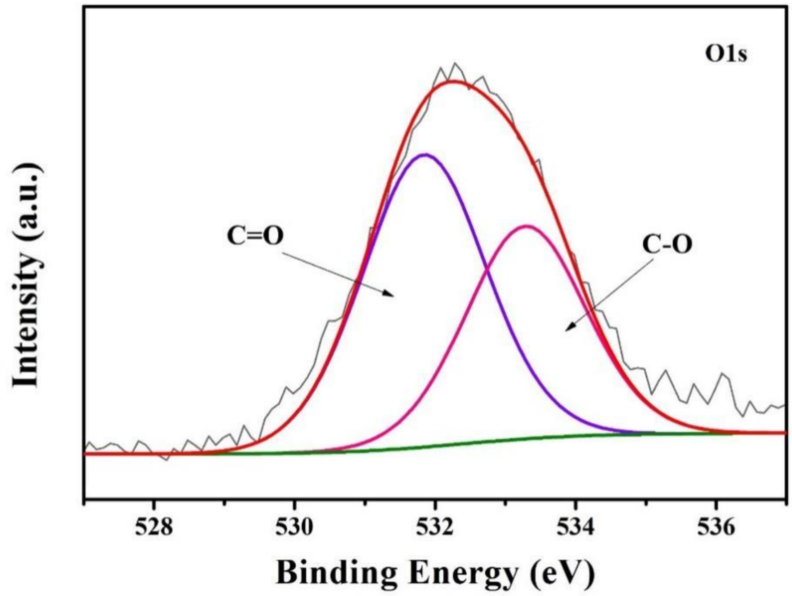

Figure S11. High-resolution XPS O1s spectrum of BPCFs.

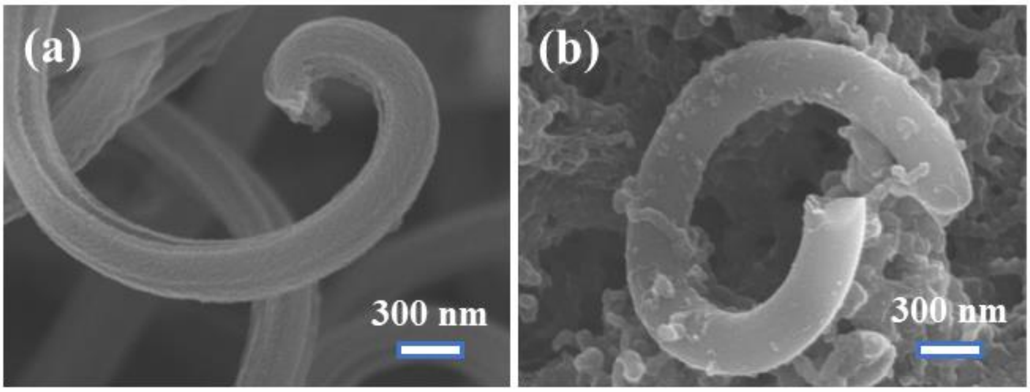

Figure S12. SEM images of BPCFs (a) before the cycle, (b) after 500 cycles.

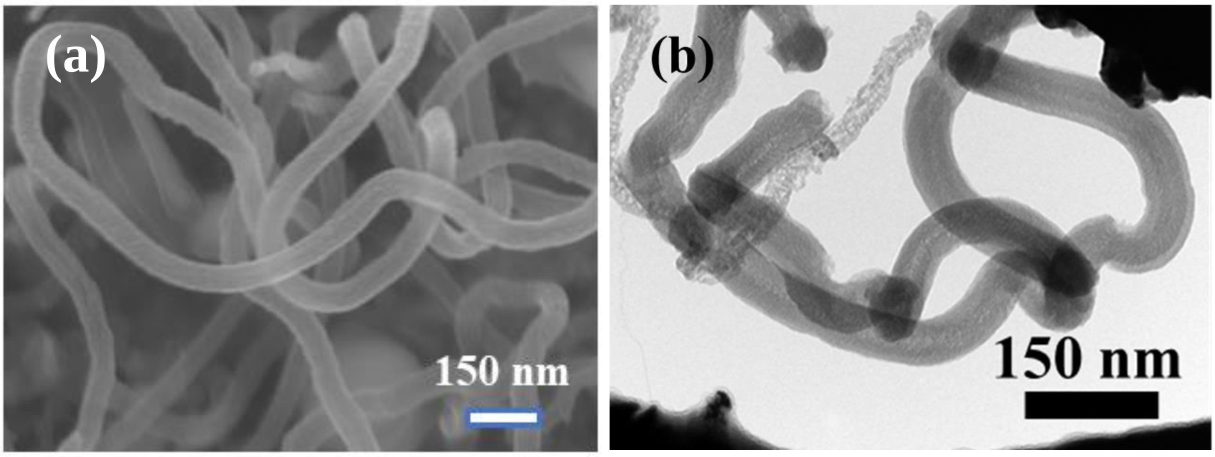

Figure S13. The morphology of SWCFs: a) SEM image and b) TEM image.

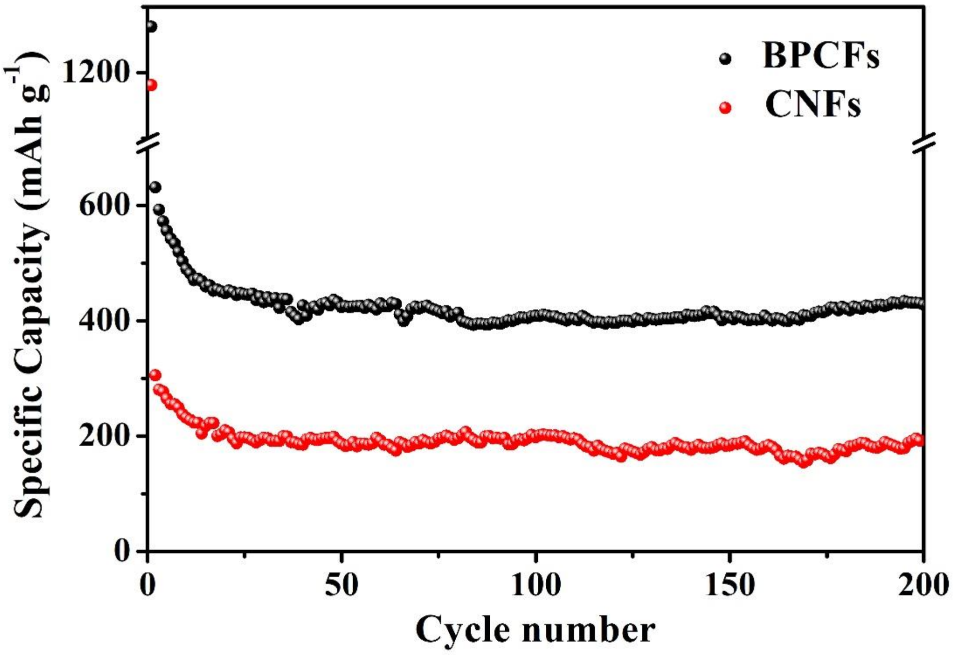

Figure S14. Cycling performance of BPCFs and SWCFs at a current density of 0.1 A g^{-1}.

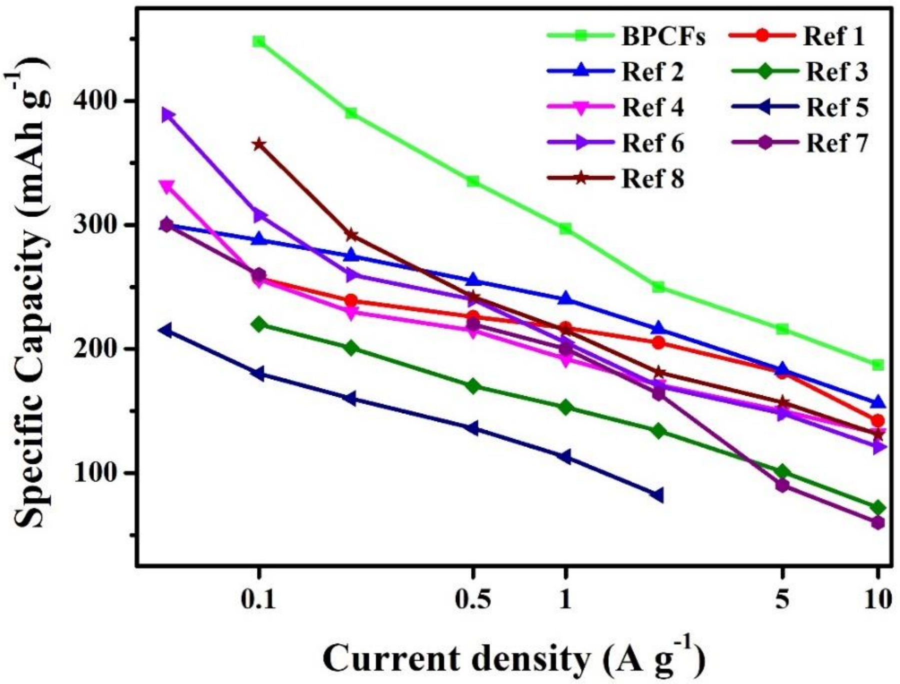

Figure S15. Rate performances of BPCFs and other reported carbon materials.

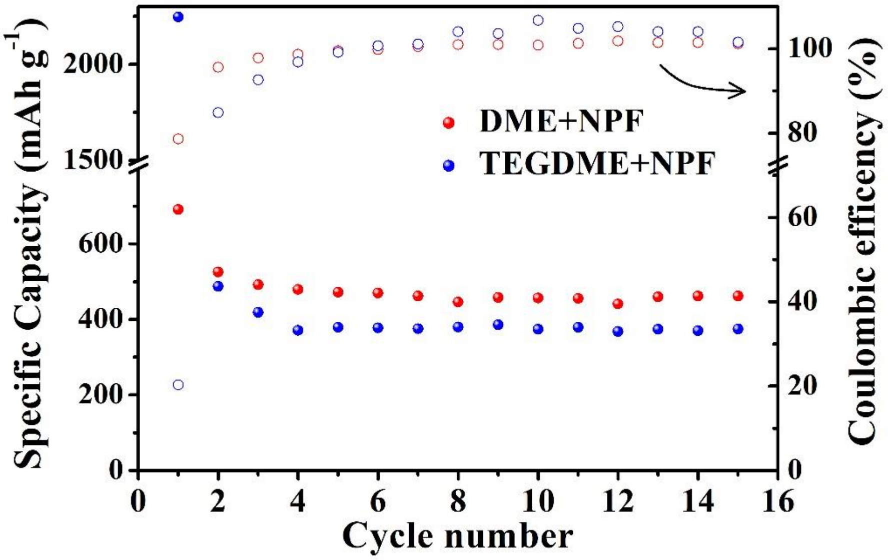

Figure S16. Cycling performance of BPCFs at a current density of 0.1 A g^{-1}. As well as using 1 M $NaPF_6$ (NPF) in a Dimethoxyethane (DME) and Tetraethylene glycol dimethyl ether

(TEGDME) as the electrolytes, respectively.

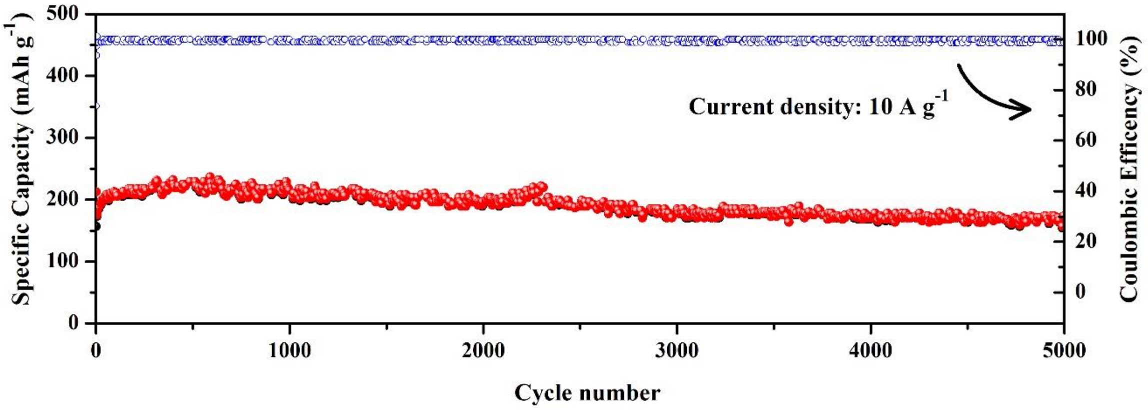

Figure S17. Long-term cycling performance of BPCFs at 10A g^{-1} with 1 mg cm^{-2}.

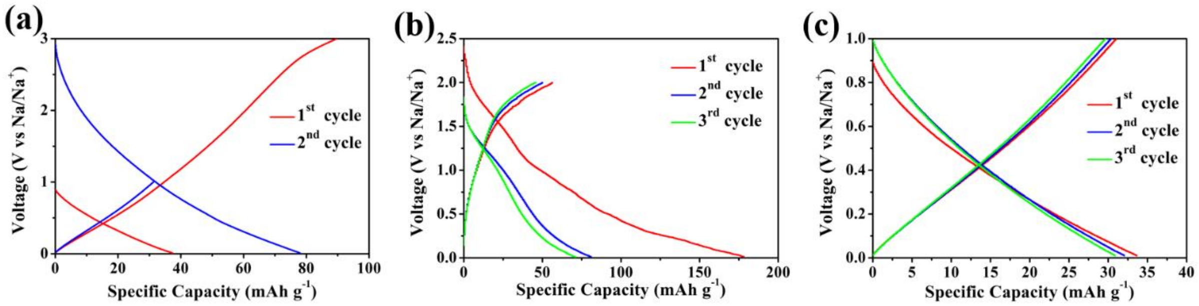

Figure S18. Discharge/charge voltage profiles of BPCFs||BPCFs symmetric cells in different voltage ranges (a) 0-3 V, (b) 0-2 V, (c) 0-1 V.

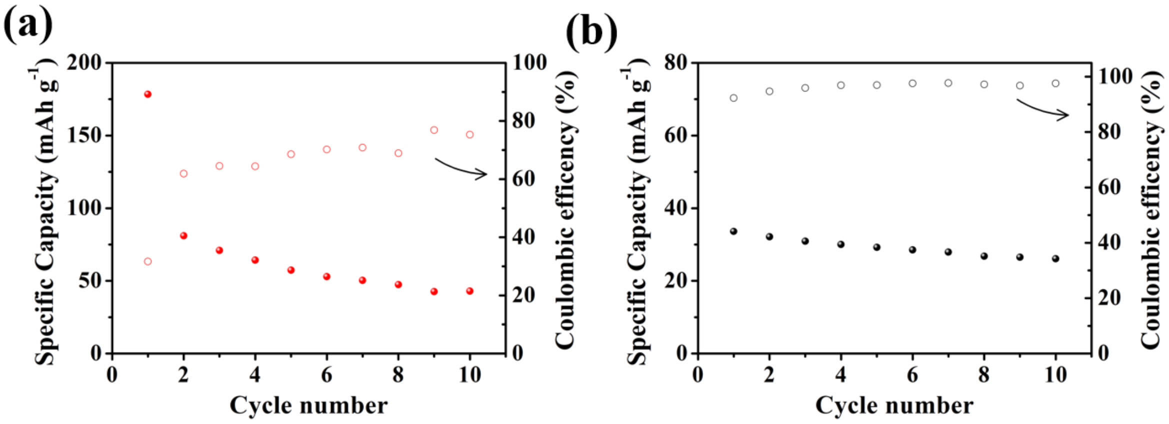

Figure S19. Cycling performance of BPCFs||BPCFs symmetric cells used 1 M $NaClO_4$/EC/DEC/FEC electrolyte in different voltage ranges (a) 0-2 V, (b) 0-1 V.

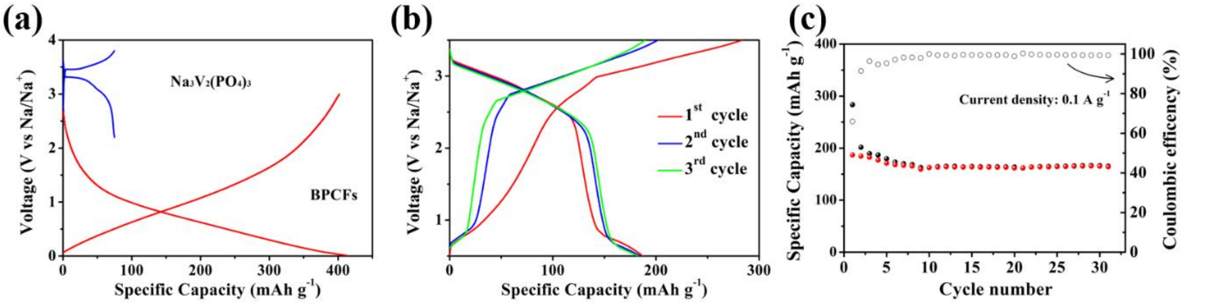

Figure S20. (a) Charge and discharge curves of the Na_3V_2 $(PO_4)_3$ (NVP) and BPCFs electrodes used 1 M $NaClO_4$/EC/DEC/FEC electrolyte at a current rate of 0.1 A g^{-1}, b) and the assembled BPCFs/NVP full cell at 0.1 A g^{-1}. c) Cycle performance of the full cell at 0.1 A g^{-1}.

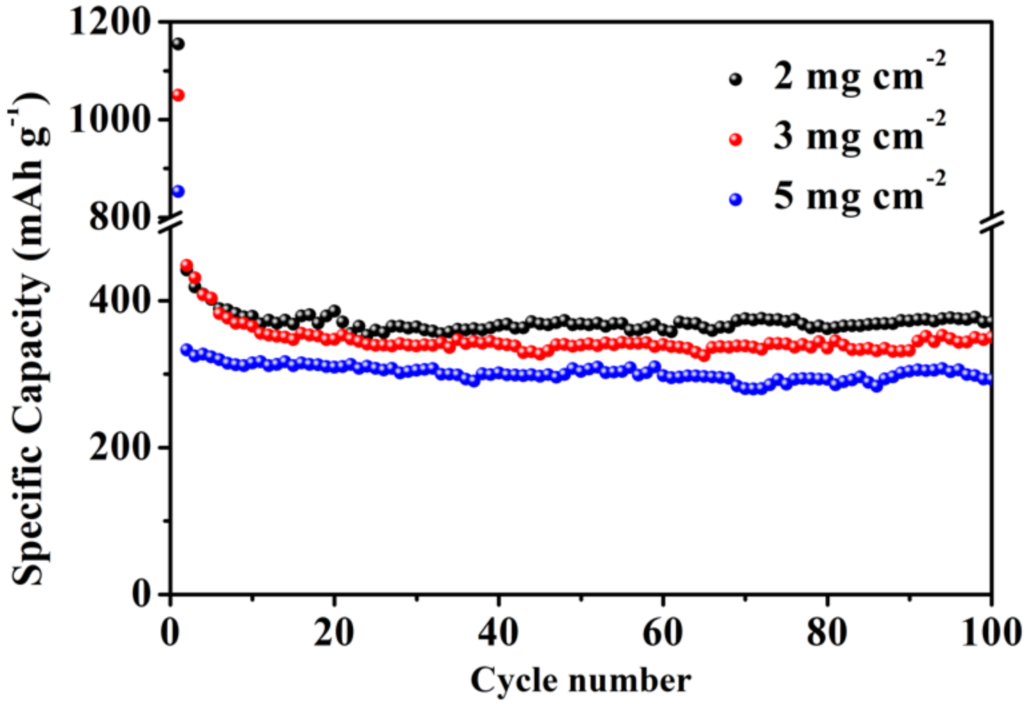

Figure S21. Cycling performance of BPCFs with different mass loading (2 mg cm^{-2}, 3 mg cm^{-2}, 5 mg cm^{-2}) at a current density of 0.1 A g^{-1}.

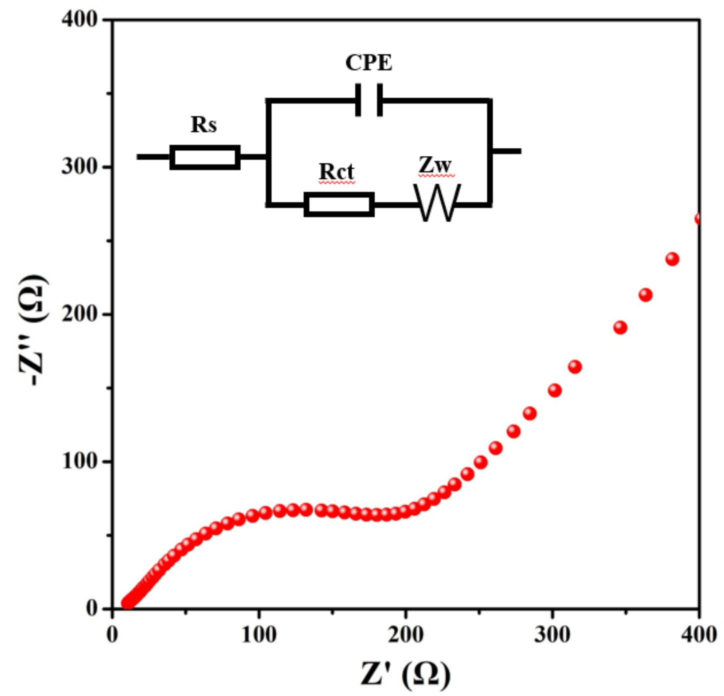

Figure S22. Nyquist plots of BPCFs anodes after cycling 5 cycles at frequencies from 100 kHz to10 MHz. The equivalent circuit model is shown in the inset.

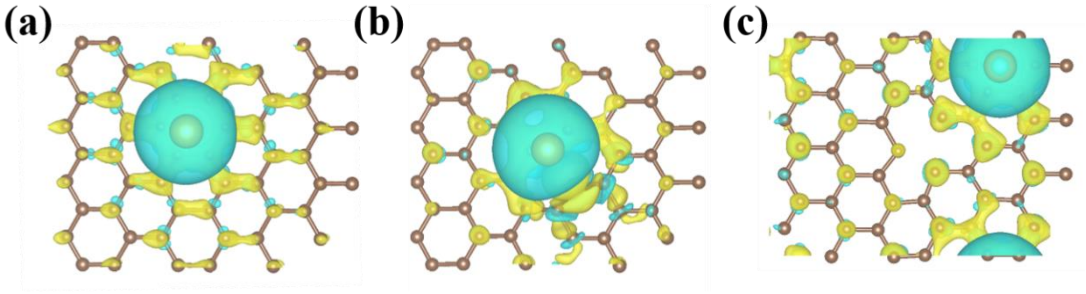

Figure S23. (a-c) Top view of the difference in electron density of Na absorbed by different carbon structures. The yellow and blue regions represent the increased and decreased electron density, respectively. The brown and yellow balls represent the C and Na atoms, respectively.

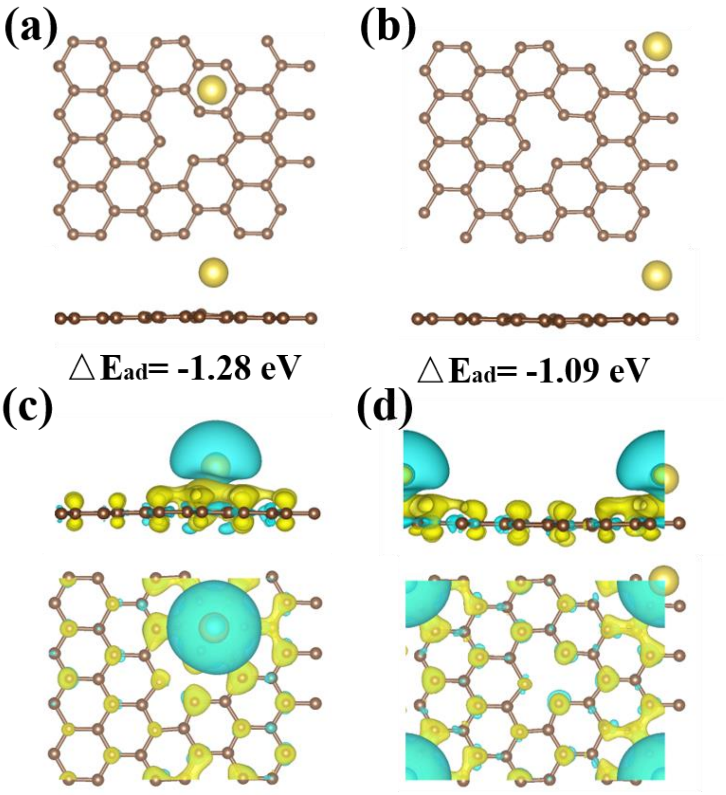

Figure S24. The simulation and adsorption site of a Na atom on the (a) ideas carbon structures (b) single vacancy defect. (c and d) Side view and top view of the different charge density of Na absorbed by different carbon structures. The yellow and blue regions represent charge accumulation and depletion, respectively. The brown and yellow balls represent the C and Na atoms, respectively.

Table S1. Elemental atomic analysis of the $Cu_{30}Mn_{70}$ alloy and nano-porous copper (NPC).

	Mn/$_{At\%}$	Cu/$_{At\%}$	O/$_{At\%}$
$Cu_{30}Mn_{70}$ alloy	69.31	30.69	0
NPC	2.35	92.41	5.24

Table S2. Distribution of C species obtained from the deconvolution of the C1s peaks.

Sample	sp^2/$_{\%}$	sp^3/$_{\%}$	C-O/$_{\%}$	C=O/$_{\%}$
BPCFs	53.2	30.8	9.6	6.4

Table S3. Electrochemical properties comparison of carbon materials reported in literature.

Samples	Specific capacity	Cycle performance	Loading	Reference
Braided porous carbon fibres (BPCFs)	400 mA h g^{-1} at 0.1A g^{-1}	173 mA h g^{-1} at 10A g^{-1} over 5000 cycles	1.0 mg cm^{-2}	This work
Carbon nanotubes on nickel foam	258 mA h g^{-1} at 0.1A g^{-1}	134 mA h g^{-1} at 10A g^{-1} over 500 cycles	1.0 mg cm^{-2}	S1
DT-C	288 mA h g^{-1} at 0.1A g^{-1}	153 mA h g^{-1} at 10A g^{-1} over 5000 cycles	-	S2
Three-dimensional porous carbon	243 mA h g^{-1} at 0.1A g^{-1}	120 mA h g^{-1} at 5A g^{-1} over 1000 cycles	0.8 mg cm^{-2}	S4
Porous carbon nanofibres	266 mA h g^{-1} at 50 mA g^{-1}	140 mA h g^{-1} at 0.5A g^{-1} over 1000 cycles	1.0 mg cm^{-2}	S7
Reduced holey graphene oxide	365 mA h g^{-1} at 0.1A g^{-1}	163 mA h g^{-1} at 2 A g^{-1} after 3000 cycles	1.23 mg cm^{-2}	S8
The sandwich-like hierarchically porous carbon/graphene	400 mA h g^{-1} at 50 mA g^{-1}	250 mA h g^{-1} at 1 A g^{-1} over 1000 cycles	-	S9
Three-dimensional hard carbon matrix	233 mA h g^{-1} at 50 mA g^{-1}	116 mA h g^{-1} at 4 A g^{-1} after 3000cycles	1.0 mg cm^{-2}	S10
Carbon nanofibres	255 mA h g^{-1} at 40 mA g^{-1}	176 mA h g^{-1} at 0.2A g^{-1} over 600 cycles	1.1 mg cm^{-2}	S11
Porous carbon nanotubes	337 mA h g^{-1} at 0.1A g^{-1}	110 mA h g^{-1} at 5 A g^{-1} after 1200 cycles	-	S12
Carbon nanofiber@ nitrogen-doped porous carbon	240 mA h g^{-1} at 0.1A g^{-1}	149 mA h g^{-1} at 0.5A g^{-1} over 400 cycles	-	S13
Nitrogen-doped carbon nanofiber film	377 mA h g^{-1} at 0.1A g^{-1}	210 mA h g^{-1} at 5 A g^{-1} after 7000 cycles	0.64 mg cm^{-2}	S14
Graphene monolayers or bilayers highly-scattered in	408.8 mA h g^{-1} at 0.1A g^{-1}	301mA h g^{-1} at 2 A g^{-1} after 1000 cycles	1.2 mg cm^{-2}	S15

porous carbon nanofibres		

Table S4. electrochemical impedance comparison of carbon materials reported in the literature.

Samples	The charge transfer resistance (R_{ct})/Ω	Reference
braided porous carbon fibres (BPCFs)	190	this work
carbon nanofibres	447.5	S5
The sandwich-like hierarchically porous carbon/graphene	415	S9
3D N-doped graphene foams	502	S16
Multi-shelled hollow hard carbon nanospheres	256	S17

Reference:

[1] W. Han, D. Chen, Q. Li, W. Liu, H. Chu, X. Rui, *J. Power Sources* **2019**, *439*, 227072.

[2] R. Guo, C. Lv, W. Xu, J. Sun, Y. Zhu, X. Yang, J. Li, J. Sun, L. Zhang, D. Yang, *Adv. Energy Mater* **2020**, *10*, 1903652.

[3] L. Fu, K. Tang, K. Song, P. A. van Aken, Y. Yu, J. Maier, *Nanoscale* **2014**, *6*, 1384.

[4] C. Zhou, D. Wang, A. Li, E. Pan, H. Liu, X. Chen, M. Jia, H. Song, *Chem. Eng. J.* **2020**, *380*, 122457.

[5] T. Chen, Y. Liu, L. Pan, T. Lu, Y. Yao, Z. Sun, D. H. Chua, Q. Chen, *J. Mater.Chem. A* **2014**, *2*, 4117.

[6] X. Guo, X. Zhang, H. Song, J. Zhou, *J. Mater.Chem. A* **2017**, *5*, 21343.

[7] W. Li, L. Zeng, Z. Yang, L. Gu, J. Wang, X. Liu, J. Cheng, Y. Yu, *Nanoscale* **2014**, *6*, 693.

[8] J. Zhao, Y. Z. Zhang, F. Zhang, H. Liang, F. Ming, H. N. Alshareef, Z. Gao, *Adv. Energy Mater* **2019**, *9*, 1803215.

[9] Y. Yan, Y. Yin, Y. Guo, L. Wan, *Adv. Energy Mater* **2014**, *4*, 1301584.

[10] Z. Yuan, L. Si, X. Zhu, *J. Mater.Chem. A* **2015**, *3*, 23403.

[11] W. Luo, J. Schardt, C. Bommier, B. Wang, J. Razink, J. Simonsen, X. L. Ji, *J. Mater.Chem. A* **2013**, *1*, 10662.

[12] Z. Chen, T. Wang, M. Zhang, G. Cao, *Small* **2017**, *13*, 1604045.

[13] Z. Zhang, J. Zhang, X. Zhao, F. Yang, *Carbon* **2015**, *95*, 552.

[14] S. Wang, L. Xia, L. Yu, L. Zhang, H. Wang, X. Lou, *Adv. Energy Mater* **2016**, *6*, 1502217.

[15] Y. Liu, L. Fan, L. Jiao, *J. Mater.Chem. A* **2017**, *5*, 1698.

[16] J. Xu, M. Wang, N. P. Wickramaratne, M. Jaroniec, S. Dou, L. Dai, *Adv. Mater.* **2015**, *27*, 2042.

[17] D. Bin, Y. Li, Y. Sun, S. Duan, Y. Lu, J. Ma, A. Cao, Y. Hu, L. Wan, *Adv. Energy Mater* **2018**, *8*, 1800855.
